# Associations of systemic inflammatory indices with rheumatoid arthritis and all-cause mortality: a NHANES study with independent hospital-based replication

**DOI:** 10.3389/fmed.2026.1968785

**Published:** 2026-10-02

**Authors:** Lin Wang, Hao Yu, Yuanyuan Cao, Li Yu

**Affiliations:** 1 Department of Microbiology and Parasitology, Anhui Provincial key Laboratory of Zoonoses, School of Basic Medical Sciences, Anhui Medical University, Hefei, Anhui, China; 2 Department of Clinical Laboratory, The First Affiliated Hospital of Anhui University of Chinese Medicine, Hefei, Anhui, China

**Keywords:** all-cause mortality, CALLY, inflammatory indices, RAR, rheumatoid arthritis, systemic inflammation

## Abstract

**Background:**

Rheumatoid arthritis (RA) is a chronic systemic inflammatory disease associated with substantial morbidity and increased mortality. Although several composite inflammatory indices have been proposed as accessible biomarkers, their associations with RA and long-term prognosis remain incompletely understood.

**Methods:**

We analyzed 15,545 adults from five NHANES cycles (2001–2010), including 1,075 participants with self-reported RA. Survey-weighted multivariable logistic regression was used to assess associations between inflammatory indices and prevalent RA. Among participants with RA, survey-weighted Kaplan–Meier and Cox regression analyses were used to evaluate all-cause mortality. Restricted cubic spline analyses examined potential nonlinear associations. An independent retrospective hospital-based cohort was used to assess the directional consistency of key RA-related associations.

**Results:**

Among 15,545 participants, 1,075 had self-reported RA. In the fully adjusted models, participants in the highest RAR quartile had greater odds of prevalent self-reported RA than those in the lowest quartile (OR, 1.86; 95% CI, 1.33–2.60), whereas higher CALLY was inversely associated with RA (Q4 vs. Q1: OR, 0.58; 95% CI, 0.43–0.78); both associations remained significant after FDR correction. Among participants with self-reported RA, higher RAR was associated with a greater hazard of all-cause mortality (Q4 vs. Q1: HR, 2.48; 95% CI, 1.62–3.77), whereas higher CALLY was associated with a lower mortality hazard (Q4 vs. Q1: HR, 0.44; 95% CI, 0.31–0.61), with both associations remaining significant after FDR correction. Restricted cubic spline analyses suggested nonlinear associations for several inflammatory indices. In the independent hospital-based cohort, several key RA-related inflammatory indices showed directionally consistent associations, supporting the reproducibility of the principal RA-related findings.

**Conclusion:**

RAR and CALLY showed the most consistent associations with both prevalent RA and all-cause mortality. These accessible inflammatory indices may warrant further evaluation as markers of systemic inflammatory and nutritional status in RA, but prospective validation is needed before clinical application.

## Introduction

Rheumatoid arthritis (RA) is a chronic autoimmune disease characterized by persistent synovial inflammation, progressive joint destruction, and systemic complications (1–3). It affects approximately 0.5% of the global population and is associated with substantial morbidity, disability, and increased mortality (3). In addition to articular damage, RA is increasingly recognized as a systemic inflammatory disorder that contributes to the development of cardiovascular disease, metabolic abnormalities, and other comorbidities, thereby significantly reducing life expectancy (4–6). Accurate assessment of disease-related systemic inflammation and its association with clinical outcomes is therefore of considerable clinical importance.

Systemic inflammation plays a central role in the pathogenesis and progression of RA (7, 8). In recent years, a variety of hematological and biochemical composite indices, such as the neutrophil-to-lymphocyte ratio (NLR), systemic immune-inflammation index (SII), and systemic inflammation response index (SIRI), have been proposed as convenient and cost-effective biomarkers reflecting immune and inflammatory status (9–11). More recently, novel indices integrating nutritional and inflammatory components, including the red cell distribution width-to-albumin ratio (RAR) and CALLY index, have gained attention for their potential prognostic value (12, 13). Although these markers have been widely investigated in cancer and cardiovascular diseases, their roles in RA, particularly in relation to prevalent self-reported RA and long-term outcomes, remain incompletely understood. Moreover, most previous studies have focused on single markers rather than systematically evaluating multiple inflammatory indices within a unified analytical framework.

Therefore, the present study aimed to comprehensively investigate the associations between multiple systemic inflammatory indices and RA using a large, nationally representative dataset from NHANES. In addition, an independent hospital-based cohort was used to assess the reproducibility of the principal RA-related associations observed in NHANES. These findings may provide additional insights into the clinical relevance of inflammatory biomarkers in RA and their potential value for disease assessment and prognosis.

## Methods

### Study population and data sources

This study used data from five consecutive 2-year cycles of the National Health and Nutrition Examination Survey (NHANES), spanning 2001–2002–2009–2010. The primary analytic sample included adults aged 20 years or older with valid self-reported arthritis and RA information, complete laboratory data required to calculate the inflammatory indices, and complete data on the prespecified covariates.

For the analysis of prevalent RA, participants with self-reported RA were compared with participants without self-reported arthritis. Participants with missing or indeterminate arthritis/RA status, missing laboratory measurements required for inflammatory-index calculation, or missing prespecified covariates were excluded. The final cross-sectional analytic sample comprised 15,545 participants, including 14,470 participants without self-reported arthritis and 1,075 participants with self-reported RA. The fully adjusted analysis of prevalent self-reported RA was based on 15,532 participants with complete data for all variables included in Model 3. The complete participant-selection process is presented in Supplementary Figure S1. Variable-specific missingness before complete-case selection is presented in Supplementary Table S1.

For the mortality analysis, the cohort was restricted to the 1,075 participants with self-reported RA who had available survival status and follow-up time. During follow-up, 376 deaths occurred. To account for the complex multistage sampling design of NHANES, all analyses incorporated sampling weights, strata, and primary sampling units. When pooling the five survey cycles, the 2-year MEC examination weight was divided by five to create the 10-year examination weight.

An independent retrospective hospital-based cohort was used to replicate the principal associations between inflammatory indices and RA observed in NHANES. Adults aged 18 years or older who attended the participating hospital between January 2023 and August 2026 were eligible. The cohort included patients with RA and health-examination participants without RA. RA diagnoses were ascertained from hospital medical records and had been established by rheumatologists during routine clinical care. Health-examination participants were required to have no documented history of RA or other autoimmune diseases. Participants were excluded if key demographic data were missing, laboratory measurements were insufficient to calculate the inflammatory indices, duplicate records were identified, or severe acute infection, hematologic disease, malignancy, or other conditions likely to substantially influence systemic inflammatory markers were present. For participants with repeated records, laboratory measurements closest to the index date were retained, defined as the date of RA diagnosis for patients with RA and the examination date for health-examination participants. Inflammatory indices were calculated using the same definitions as in the NHANES analysis, and covariates were harmonized where available. Analyses of the hospital cohort were conducted using methods appropriate for a retrospective, non-probability clinical cohort; NHANES sampling weights and design variables were not applied. The hospital-based component of this study was approved by the Ethics Committee of the First Affiliated Hospital of Anhui University of Chinese Medicine (2026AH-129-01). Owing to the retrospective nature of the study, the requirement for written informed consent was waived.

### Definition of rheumatoid arthritis and mortality outcome

In NHANES, RA status was defined using self-reported physician-diagnosed arthritis and the reported type of arthritis. Participants who reported having been told by a physician or other health professional that they had arthritis and identified rheumatoid arthritis as the arthritis type were classified as having self-reported RA. Participants who reported no physician-diagnosed arthritis were classified as participants without self-reported RA. Participants who reported other forms of arthritis or whose arthritis type could not be determined were excluded from the RA/non-RA classification.

In the hospital-based replication cohort, RA cases were identified from the hospital information system based on a documented clinical diagnosis established by a rheumatologist during routine clinical care. Non-RA health-examination participants were selected from individuals undergoing routine health examinations who had no documented history of RA or other autoimmune or systemic inflammatory diseases. For participants with repeated laboratory records, the measurements obtained closest to the date of RA diagnosis were retained.

All-cause mortality was evaluated only in the NHANES cohort and was ascertained through linkage to the National Death Index. Mortality status and follow-up time were obtained from the NHANES 2019 public-use Linked Mortality File, with follow-up through December 31, 2019. Follow-up time was calculated from the baseline NHANES examination to death or the end of follow-up, whichever occurred first.

### Inflammatory indices assessment

Routine hematological and biochemical parameters were used to calculate systemic inflammatory indices. Before calculation, laboratory measurements from the NHANES and hospital-based cohorts were harmonized to consistent units. Neutrophil, lymphocyte, monocyte, and platelet counts were expressed as  × 10⁹/L; serum albumin as g/L; C-reactive protein (CRP) as mg/L; red blood cell distribution width (RDW) as a percentage; and total bilirubin as μmol/L.

The inflammatory indices were calculated as follows (14): systemic inflammation response index (SIRI) = neutrophil count×monocyte count/lymphocyte count; C-reactive protein–albumin–lymphocyte index (CALLY) = albumin×lymphocyte count/(CRP×10) (15); red blood cell distribution width-to-albumin ratio (RAR) = RDW/albumin; neutrophil-to-lymphocyte ratio (NLR) = neutrophil count/lymphocyte count; platelet-to-lymphocyte ratio (PLR) = platelet count/lymphocyte count; lymphocyte-to-monocyte ratio (LMR) = lymphocyte count/monocyte count; systemic immune-inflammation index (SII) = platelet count×neutrophil count/lymphocyte count; pan-immune-inflammation value (PIV) = neutrophil count×monocyte count×platelet count/lymphocyte count; and neutrophil-to-albumin ratio (NAR) = neutrophil count/albumin. Total bilirubin was also evaluated as an inflammation-related biochemical marker.

### Covariate definition

Demographic and socioeconomic variables included age, sex, race/ethnicity, education level, marital status, and poverty income ratio (PIR). Lifestyle factors included smoking status and alcohol consumption. Clinical variables included BMI, total cholesterol, HbA1c, diabetes, hypertension, heart disease, coronary heart disease, stroke, and cancer.

### Statistical analysis

All analyses were conducted using R software (version 4.4.2). For NHANES analyses, the complex, multistage probability sampling design was accounted for by incorporating examination sample weights, strata, and primary sampling units. Data from five 2-year NHANES cycles (2001–2010) were pooled, and 10-year examination weights were calculated by dividing the original 2-year MEC examination weight by five. Survey designs were specified using NHANES strata and primary sampling unit variables, with nesting of primary sampling units within strata. Variance estimates were obtained using Taylor series linearization.

Descriptive characteristics are presented as unweighted sample sizes together with survey-weighted estimates. Continuous variables are reported as survey-weighted means with standard errors (SEs), and categorical variables are reported as unweighted counts and survey-weighted percentages with SEs. Between-group comparisons were conducted using design-based linear regression for continuous variables and Rao–Scott chi-square tests for categorical variables.

### Association between inflammatory indices and rheumatoid arthritis

Survey-weighted multivariable logistic regression models were used to evaluate associations between inflammatory indices and self-reported RA. Inflammatory indices were categorized into survey-weighted quartiles, with the lowest quartile serving as the reference category. Odds ratios (ORs) and 95% confidence intervals (CIs) were estimated using survey-weighted regression models. Outcome-specific survey-weighted quartile cutoffs are provided in Supplementary Table S2.

Three progressively adjusted models were constructed: Model 1 was unadjusted; Model 2 was adjusted for age, sex, and race/ethnicity; and Model 3 was additionally adjusted for educational attainment, poverty income ratio, marital status, smoking status, alcohol consumption, body mass index, total cholesterol, hemoglobin A1c, diabetes, hypertension, heart disease, coronary heart disease, stroke, and cancer. Tests for linear trend across quartiles were performed by modeling quartile categories as ordinal variables.

### Subgroup and interaction analyses

Prespecified subgroup analyses were conducted according to sex, age, smoking status, and alcohol consumption. Fully adjusted survey-weighted logistic regression models were fitted within each subgroup, excluding the stratification variable from the corresponding model. Effect modification was assessed by including multiplicative interaction terms between each inflammatory index and the subgroup variable. Interaction significance was evaluated using design-based Wald tests.

### Survival analyses

Among participants with RA, survey-weighted Cox regression models were used to estimate hazard ratios (HRs) and 95% CIs for the associations between inflammatory indices and all-cause mortality. The covariate-adjustment strategy for the three Cox models was identical to that used in the logistic regression analyses. Inflammatory indices were analyzed in survey-weighted quartiles, with the lowest quartile as the reference, and linear trends were assessed using ordinal quartile variables.

Survey-weighted Kaplan–Meier curves were constructed to compare survival probabilities across quartiles of selected inflammatory indices. Global differences between survival curves were evaluated using survey-weighted log-rank tests.

### Restricted cubic spline analyses

Restricted cubic spline analyses were performed to examine potential nonlinear associations between inflammatory indices and RA or all-cause mortality. Survey-weighted logistic regression models were used for RA, whereas survey-weighted Cox regression models were used for mortality among participants with RA. Each inflammatory index was modeled as a continuous variable using restricted cubic splines with knots placed at the survey-weighted 5th, 35th, 65th, and 95th percentiles. The survey-weighted median value of each index was used as the reference. All spline models were adjusted for the covariates included in Model 3. Overall and nonlinear associations were evaluated using design-based joint Wald tests, and adjusted ORs or HRs with 95% CIs were plotted across the observed distribution of each inflammatory index.

### Multiple-testing correction

All tests were two-sided. A raw *P* value <0.05 was considered nominally significant. To account for multiple comparisons, *P* values were adjusted using the Benjamini–Hochberg false discovery rate (FDR) procedure within prespecified testing families, including fully adjusted quartile contrasts, trend tests, interaction tests, weighted log-rank tests, and restricted cubic spline tests. An FDR-adjusted *P* value <0.05 was considered statistically significant for analyses subject to multiplicity correction.

The hospital-based replication cohort was analyzed separately as a retrospective, non-probability sample; therefore, NHANES sampling weights, strata, and primary sampling units were not applied. However, inflammatory-index definitions and covariate coding were harmonized as far as possible with those used in the NHANES analyses.

## Results

### Baseline characteristics of study participants

A total of 15,545 participants were included, comprising 14,470 participants without self-reported arthritis and 1,075 participants with self-reported RA (Table 1). The survey-weighted mean age was higher among participants with RA than among those without self-reported arthritis [56.62 [SE, 0.54] vs. 42.85 [SE, 0.23] years; *P* < 0.001]. The weighted proportion of females was also higher in the RA group [58.3% [SE, 2.1%] vs. 48.5% [SE, 0.4%]; *P* < 0.001]. Participants with RA had higher weighted prevalences of hypertension (52.5% vs. 23.1%), diabetes (16.5% vs. 5.3%), and heart disease (10.0% vs. 2.1%) (all *P* < 0.001). In survey-weighted descriptive comparisons, the RA group had higher levels of SIRI, RAR, NLR, PLR, SII, PIV, and NAR, and lower levels of CALLY and bilirubin (all *P* ≤ 0.010). LMR did not differ significantly between groups (*P* = 0.117). These descriptive findings indicate systematic differences in inflammatory-marker profiles between the two groups.

**Table 1:** Survey-weighted characteristics of participants with self-reported rheumatoid arthritis and participants without self-reported arthritis, NHANES 2001–2010.

Variable		No_RA	RA	*p*_value
Unweighted N		14,470	1,075	
Age	Weighted mean (SE)	42.85 (0.23)	56.62 (0.54)	<0.001
PIR	Weighted mean (SE)	3.12 (0.03)	2.57 (0.06)	<0.001
BMI	Weighted mean (SE)	28.17 (0.09)	30.53 (0.33)	<0.001
TC	Weighted mean (SE)	5.14 (0.01)	5.13 (0.03)	0.834
HbA1c	Weighted mean (SE)	5.45 (0.01)	5.78 (0.04)	<0.001
Gender	Female	7,046; 48.5% (0.4)	609; 58.3% (2.1)	<0.001
	Male	7,424; 51.5% (0.4)	466; 41.7% (2.1)	
Race	Mexican American	3,118; 9.0% (0.8)	165; 5.5% (1.0)	<0.001
	Non-Hispanic Black	2,694; 10.4% (0.7)	308; 16.9% (1.6)	
	Non-Hispanic White	7,092; 70.8% (1.4)	510; 69.7% (2.3)	
	Other Hispanic	965; 4.6% (0.6)	65; 4.3% (1.1)	
	Other Race	601; 5.2% (0.4)	27; 3.6% (0.8)	
Education	9–11th Grade	2,191; 11.2% (0.5)	224; 16.5% (1.3)	<0.001
	College Graduate or above	3,178; 28.6% (0.9)	121; 13.9% (1.4)	
	High School Grad	3,346; 23.8% (0.5)	253; 28.1% (2.1)	
	Less Than 9th Grade	1,623; 5.1% (0.3)	180; 10.0% (1.2)	
	Some College	4,132; 31.3% (0.6)	297; 31.4% (2.0)	
Marry	Divorced	1,328; 9.2% (0.3)	167; 14.5% (1.5)	<0.001
	Living with partner	1,240; 8.3% (0.4)	46; 5.2% (0.9)	
	Married	8,069; 58.2% (0.8)	552; 56.3% (2.1)	
	Never married	2,616; 18.3% (0.7)	80; 6.8% (0.9)	
	Separated	440; 2.4% (0.2)	49; 3.6% (0.6)	
	Widowed	777; 3.6% (0.2)	181; 13.5% (1.1)	
Smoke	No	7,847; 53.7% (0.8)	438; 38.9% (2.1)	<0.001
	Yes	6,623; 46.3% (0.8)	637; 61.1% (2.1)	
Alcohol	No	4,010; 23.4% (1.0)	375; 33.9% (2.2)	<0.001
	Yes	10,460; 76.6% (1.0)	700; 66.1% (2.2)	
Diabetes	Borderline	170; 1.0% (0.1)	34; 3.2% (0.6)	<0.001
	No	13,170; 93.7% (0.2)	816; 80.3% (1.7)	
	Yes	1,130; 5.3% (0.2)	225; 16.5% (1.5)	
Hypertension	No	10,724; 76.9% (0.6)	456; 47.5% (2.1)	<0.001
	Yes	3,746; 23.1% (0.6)	619; 52.5% (2.1)	
Heart	No	14,067; 97.9% (0.1)	958; 90.0% (1.1)	<0.001
	Yes	390; 2.1% (0.1)	117; 10.0% (1.1)	
CHD	No	14,062; 97.7% (0.2)	983; 92.4% (0.9)	<0.001
	Yes	408; 2.3% (0.2)	92; 7.6% (0.9)	
Stroke	No	14,181; 98.6% (0.1)	973; 91.6% (1.3)	<0.001
	Yes	289; 1.4% (0.1)	102; 8.4% (1.3)	
Cancer	No	13,517; 93.5% (0.3)	923; 84.8% (1.3)	<0.001
	Yes	953; 6.5% (0.3)	152; 15.2% (1.3)	
SIRI	Weighted mean (SE)	1.23 (0.01)	1.38 (0.04)	<0.001
CALLY	Weighted mean (SE)	9.33 (0.15)	5.41 (0.33)	<0.001
RAR	Weighted mean (SE)	0.298 (0.001)	0.320 (0.002)	<0.001
NLR	Weighted mean (SE)	2.19 (0.01)	2.37 (0.05)	<0.001
PLR	Weighted mean (SE)	135.75 (0.70)	142.23 (2.52)	0.01
LMR	Weighted mean (SE)	4.14 (0.02)	4.02 (0.07)	0.117
SII	Weighted mean (SE)	585.14 (4.23)	637.80 (15.22)	<0.001
PIV	Weighted mean (SE)	331.70 (3.24)	378.27 (14.33)	0.001
NAR	Weighted mean (SE)	0.102 (0.001)	0.110 (0.002)	<0.001
Bilirubin	Weighted mean (SE)	13.12 (0.08)	12.04 (0.16)	<0.001

Values are presented as unweighted n; survey-weighted mean for continuous variables, or unweighted n; survey-weighted percentage for categorical variables. *P* values were calculated using design-based tests and refer to the overall comparison between groups; therefore, they are shown only in the first row of each categorical variable. Variable-specific unweighted *n* may vary slightly because of missing data. Borderline diabetes indicates self-reported prediabetes/borderline diabetes.

### Survey-weighted multivariable logistic regression analysis of the associations between inflammatory indices and prevalent rheumatoid arthritis

Survey-weighted multivariable logistic regression analyses were conducted to evaluate the associations between inflammatory-index quartiles and prevalent RA across three progressively adjusted models (Figure 1). In the unadjusted model, higher quartiles of RAR, SIRI, NLR, SII, PIV, and NAR were associated with higher odds of RA, whereas higher CALLY and bilirubin quartiles were associated with lower odds of RA. These associations were generally attenuated after adjustment for age, sex, and race/ethnicity in Model 2. In the fully adjusted Model 3, higher CALLY remained inversely associated with RA across Q2, Q3, and Q4 compared with Q1; the Q4-versus-Q1 OR was 0.58 (95% CI, 0.43–0.78; FDR-adjusted *P* = 0.004). Higher RAR was associated with increased odds of RA in Q4 versus Q1 (OR, 1.86; 95% CI, 1.33–2.60; FDR-adjusted *P* = 0.004). Although higher bilirubin was nominally associated with lower odds of RA in Model 3 (Q4 vs. Q1: OR, 0.71; 95% CI, 0.55–0.93; unadjusted *P* = 0.012), this association did not remain significant after false-discovery-rate correction (adjusted *P* = 0.075). No fully adjusted associations for SIRI, NLR, PLR, LMR, SII, PIV, or NAR remained significant after FDR correction. Complete ORs, 95% CIs, unadjusted *P* values, and FDR-adjusted *P* values for all quartile contrasts and models are presented in Supplementary Table 4. The principal findings were unchanged in the parsimonious sensitivity analysis. After adjustment for age, sex, race/ethnicity, education, poverty income ratio, smoking, alcohol consumption, and body mass index, higher CALLY remained inversely associated with prevalent self-reported RA (Q4 vs. Q1: OR, 0.57; 95% CI, 0.42–0.76; FDR-adjusted *P* = 0.002), whereas higher RAR remained positively associated with prevalent self-reported RA (Q4 vs. Q1: OR, 1.94; 95% CI, 1.39–2.70; FDR-adjusted *P* = 0.002; Supplementary Figure S2).

**Figure 1:**
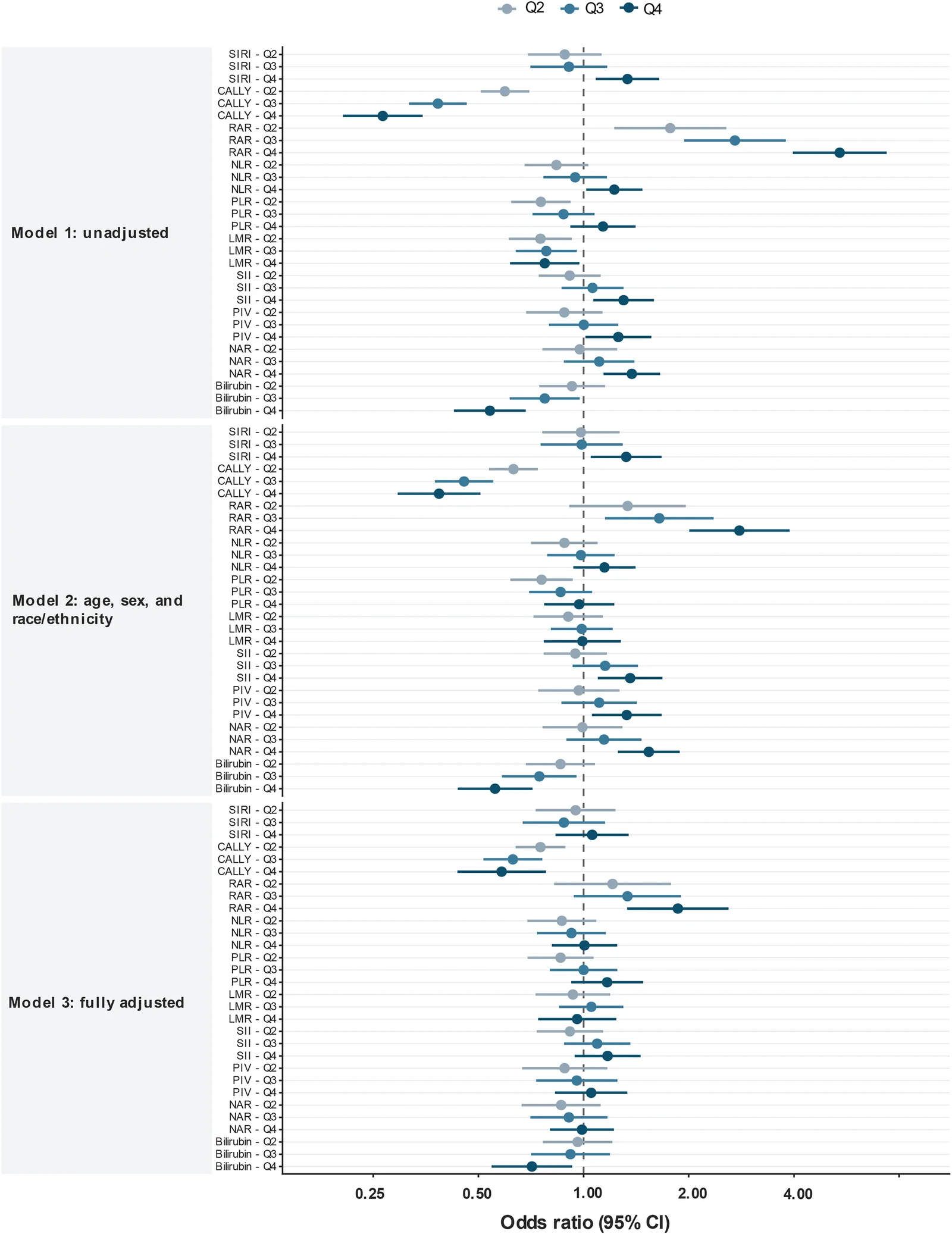
Survey-weighted associations between inflammatory-index quartiles and prevalent rheumatoid arthritis. Logistic regression models with sequential adjustment: Model 1 was unadjusted; Model 2 was adjusted for age, sex, and race/ethnicity; and Model 3 was further adjusted for education level, poverty income ratio, marital status, smoking status, alcohol consumption, body mass index, total cholesterol, HbA1c, diabetes, hypertension, heart disease, coronary heart disease, stroke, and cancer.

In exploratory fully adjusted survey-weighted restricted cubic spline analyses, RAR, CALLY, and total bilirubin were each associated overall with prevalent RA (all FDR-adjusted *P* for overall association < 0.001; Supplementary Figure S3).

### Subgroup analysis

Exploratory subgroup analyses were performed using fully adjusted survey-weighted logistic regression models (Figure 2; Supplementary Table S5). After Benjamini-Hochberg FDR correction across the 12 interaction tests, significant effect modification was observed for the association between RAR and RA by smoking status (FDR-adjusted *P* for interaction = 0.009), and for the association between bilirubin and RA by sex (FDR-adjusted *P* for interaction = 0.009) and alcohol consumption (FDR-adjusted *P* for interaction = 0.039). For RAR, the Q4-versus-Q1 association was more pronounced among non-smokers (OR, 2.66; 95% CI, 1.57–4.52) than among smokers (OR, 1.54; 95% CI, 1.02–2.34). No evidence of effect modification was observed for CALLY after FDR correction. Likewise, interactions by age were not statistically significant after correction for any of the three indices.

**Figure 2:**
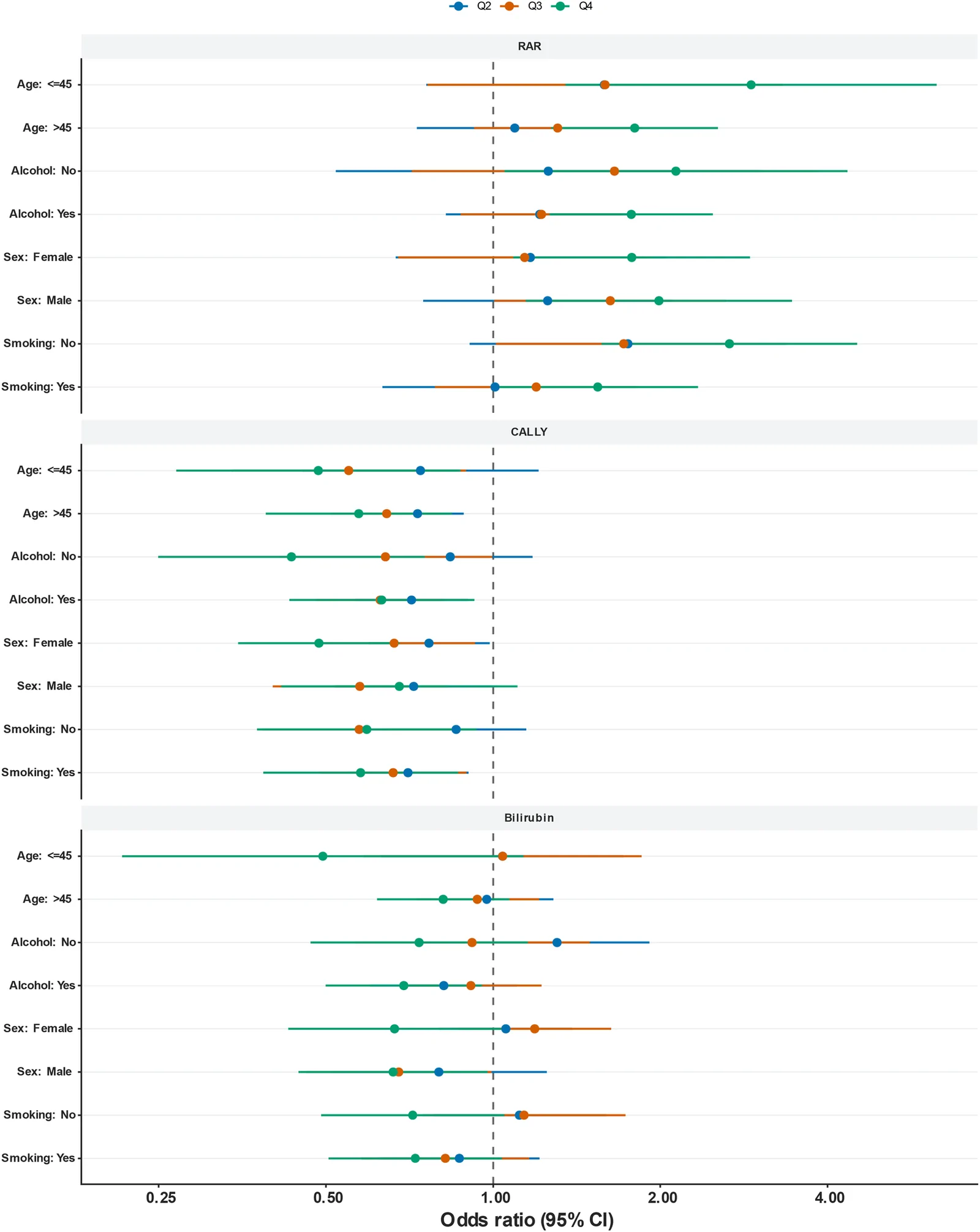
Subgroup and interaction analyses of bilirubin, CALLY, and RAR in relation to prevalent rheumatoid arthritis. Stratified associations of total bilirubin, CALLY, and RAR with RA across different subgroups. Interaction *P* values were derived from survey-weighted interaction models and were corrected for multiple testing using the Benjamini-Hochberg false-discovery-rate procedure.

### Baseline characteristics of RA patients stratified by survival status

Among the 1,075 participants with self-reported RA, 376 deaths occurred during a survey-weighted median follow-up of 167 months (13.9 years), estimated using the reverse Kaplan–Meier method (Table 2). Compared with survivors, deceased participants were older and were more likely to be male. They also had lower PIR and BMI, higher HbA1c levels, and a greater prevalence of diabetes, hypertension, heart disease, coronary heart disease, stroke, and cancer. Significant differences were also observed in race/ethnicity, educational attainment, and marital status, whereas smoking status, alcohol consumption, and total cholesterol did not differ significantly between groups. For inflammatory indices, deceased participants had higher survey-weighted mean levels of SIRI, RAR, NLR, PLR, SII, PIV, NAR, and bilirubin, but lower CALLY and LMR levels than survivors (all *P* < 0.05). These descriptive findings suggest that a greater inflammatory burden and poorer immune–nutritional status were associated with deceased survival status among participants with RA.

**Table 2:** Baseline characteristics of patients with rheumatoid arthritis according to survival status.

Characteristic	Level	Alive	Dead	*P* value
Unweighted N		699	376	
Age	Weighted mean (SE)	51.77 (0.55)	67.54 (0.76)	<0.001
PIR	Weighted mean (SE)	2.73 (0.08)	2.19 (0.08)	<0.001
BMI	Weighted mean (SE)	30.90 (0.39)	29.71 (0.52)	0.038
TC	Weighted mean (SE)	5.16 (0.04)	5.05 (0.07)	0.168
HbA1c	Weighted mean (SE)	5.72 (0.05)	5.91 (0.06)	0.013
Gender	Female	426; 61.0% (2.6)	183; 52.3% (3.2)	0.025
	Male	273; 39.0% (2.6)	193; 47.7% (3.2)	
Race	Mexican American	133; 6.8% (1.2)	32; 2.5% (0.8)	0.008
	Non-Hispanic Black	211; 18.2% (2.0)	97; 14.1% (2.1)	
	Non-Hispanic White	286; 66.1% (2.7)	224; 77.9% (3.1)	
	Other Hispanic	51; 5.1% (1.3)	14; 2.5% (1.2)	
	Other Race	18; 3.9% (1.1)	9; 3.0% (1.1)	
Education	9–11th Grade	141; 14.6% (1.5)	83; 20.7% (2.3)	0.007
	College Graduate or above	85; 14.9% (1.8)	36; 11.8% (2.5)	
	High School Grad	154; 26.9% (2.3)	99; 31.0% (3.0)	
	Less Than 9th Grade	102; 8.8% (1.4)	78; 12.6% (1.7)	
	Some College	217; 34.8% (2.5)	80; 23.9% (2.8)	
Marry	Divorced	116; 15.1% (1.6)	51; 13.2% (2.4)	<0.001
	Living with partner	37; 5.9% (1.1)	9; 3.8% (1.6)	
	Married	380; 60.4% (2.4)	172; 47.3% (3.2)	
	Never married	59; 7.5% (1.0)	21; 5.1% (1.6)	
	Separated	39; 4.5% (0.8)	10; 1.7% (0.6)	
	Widowed	68; 6.7% (0.9)	113; 28.9% (2.7)	
Smoke	No	305; 41.3% (2.7)	133; 33.6% (3.0)	0.065
	Yes	394; 58.7% (2.7)	243; 66.4% (3.0)	
Alcohol	No	238; 32.7% (2.4)	137; 36.6% (3.2)	0.243
	Yes	461; 67.3% (2.4)	239; 63.4% (3.2)	
Diabetes	Borderline	16; 2.7% (0.8)	18; 4.1% (1.2)	0.023
	No	550; 83.3% (2.0)	266; 73.6% (3.4)	
	Yes	133; 14.0% (1.9)	92; 22.2% (2.9)	
Hypertension	No	332; 54.3% (2.6)	124; 32.1% (3.0)	<0.001
	Yes	367; 45.7% (2.6)	252; 67.9% (3.0)	
Heart	No	654; 94.4% (0.9)	304; 80.2% (2.6)	<0.001
	Yes	45; 5.6% (0.9)	72; 19.8% (2.6)	
CHD	No	667; 96.6% (0.7)	316; 82.9% (2.1)	<0.001
	Yes	32; 3.4% (0.7)	60; 17.1% (2.1)	
Stroke	No	654; 93.8% (1.4)	319; 86.7% (2.1)	0.002
	Yes	45; 6.2% (1.4)	57; 13.3% (2.1)	
Cancer	No	620; 87.6% (1.5)	303; 78.6% (2.9)	0.004
	Yes	79; 12.4% (1.5)	73; 21.4% (2.9)	
SIRI	Weighted mean (SE)	1.23 (0.04)	1.71 (0.09)	<0.001
CALLY	Weighted mean (SE)	5.88 (0.41)	4.35 (0.37)	0.002
RAR	Weighted mean (SE)	0.314 (0.002)	0.334 (0.004)	<0.001
NLR	Weighted mean (SE)	2.20 (0.04)	2.76 (0.10)	<0.001
PLR	Weighted mean (SE)	138.48 (2.66)	150.66 (5.29)	0.025
LMR	Weighted mean (SE)	4.22 (0.07)	3.58 (0.13)	<0.001
SII	Weighted mean (SE)	608.17 (18.11)	704.40 (29.19)	0.003
PIV	Weighted mean (SE)	349.75 (16.15)	442.37 (25.02)	<0.001
NAR	Weighted mean (SE)	0.108 (0.002)	0.115 (0.003)	0.032
Bilirubin	Weighted mean (SE)	11.84 (0.16)	12.49 (0.28)	0.024

Values are unweighted n; survey-weighted mean (SE) for continuous variables, or unweighted n; survey-weighted percentage (SE) for categorical variables. *P* values are from design-based group comparisons. Variable-specific *n* may differ because of missing data.

### Survey-weighted associations between inflammatory indices and all-cause mortality among participants with rheumatoid arthritis

Survey-weighted Cox proportional-hazards models were used to evaluate the associations of inflammatory-index quartiles with all-cause mortality among participants with RA across three progressively adjusted models (Figure 3; Supplementary Table S6). In unadjusted analyses, higher SIRI, RAR, NLR, PIV, and NAR levels and lower CALLY and LMR levels were associated with mortality. These associations were attenuated after covariate adjustment. In the fully adjusted Model 3, after Benjamini–Hochberg false-discovery-rate correction across the 30 quartile contrasts, only the highest quartile of RAR and CALLY remained significantly associated with all-cause mortality. Compared with the lowest quartile, participants in the highest RAR quartile had a higher mortality risk (HR = 2.48; 95% CI, 1.62–3.77; FDR-adjusted *P* = 0.001), whereas those in the highest CALLY quartile had a lower mortality risk (HR = 0.44; 95% CI, 0.31–0.61; FDR-adjusted *P* < 0.001). Nominal associations for the highest quartiles of SIRI, NLR, and NAR, as well as for intermediate CALLY and bilirubin quartiles, did not remain significant after FDR correction. PLR, LMR, SII, and PIV were not significantly associated with mortality in the fully adjusted model. In a parsimonious sensitivity analysis excluding potentially downstream cardiometabolic and cardiovascular covariates (Supplementary Figure S2), the associations of CALLY and RAR with mortality remained materially unchanged. Compared with Q1, Q4 of CALLY was associated with lower mortality (HR = 0.48; 95% CI, 0.37–0.64; FDR-adjusted *P* < 0.001), whereas Q4 of RAR was associated with higher mortality (HR = 2.49; 95% CI, 1.63–3.81; FDR-adjusted *P* < 0.001), supporting the robustness of the primary findings.

**Figure 3:**
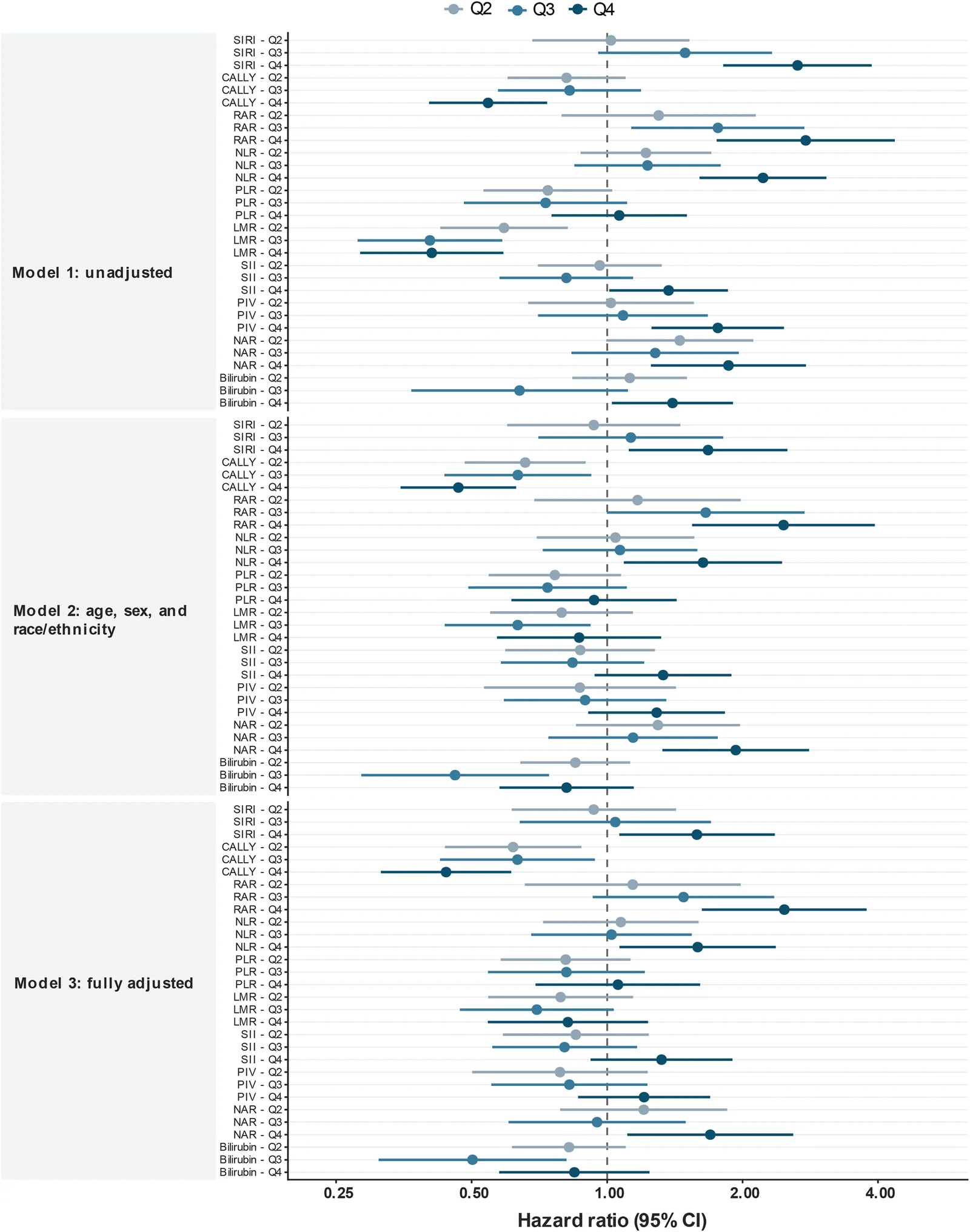
Multivariable Cox regression analyses of the associations between inflammatory indices and All- cause mortality in patients with rheumatoid arthritis. Cox regression models with sequential adjustment: Model 1 was unadjusted; Model 2 was adjusted for age, sex, and race/ethnicity; Model 3 was further adjusted for education level, poverty income ratio, marital status, smoking status, alcohol consumption, body mass index, total cholesterol, HbA1c, diabetes, hypertension, heart disease, coronary heart disease, stroke, and cancer.

Fully adjusted survey-weighted Cox subgroup analyses were conducted by age, sex, smoking status, and alcohol consumption (Supplementary Figure S4, Supplementary Table S7). After FDR correction, significant interactions were observed between CALLY and age (FDR-adjusted *P* for interaction < 0.001), all other interactions were not statistically significant.

### Survey-weighted Kaplan–Meier survival analysis of inflammatory indices

Survey-weighted Kaplan–Meier analyses demonstrated significant differences in all-cause survival across quartiles of CALLY, NLR, RAR, and SIRI among participants with RA (Figure 4; Supplementary Table S8). Higher CALLY quartiles were generally associated with more favorable survival probabilities, whereas higher quartiles of NLR, RAR, and SIRI were associated with lower survival probabilities. Global survey-weighted log-rank tests showed differences across quartiles for CALLY (*P* = 0.0002), NLR (*P* < 0.001), RAR (*P* < 0.001), and SIRI (*P* < 0.001); all remained significant after Benjamini–Hochberg FDR correction. These unadjusted survival patterns are consistent with an association between inflammatory status and all-cause mortality among participants with RA.

**Figure 4:**
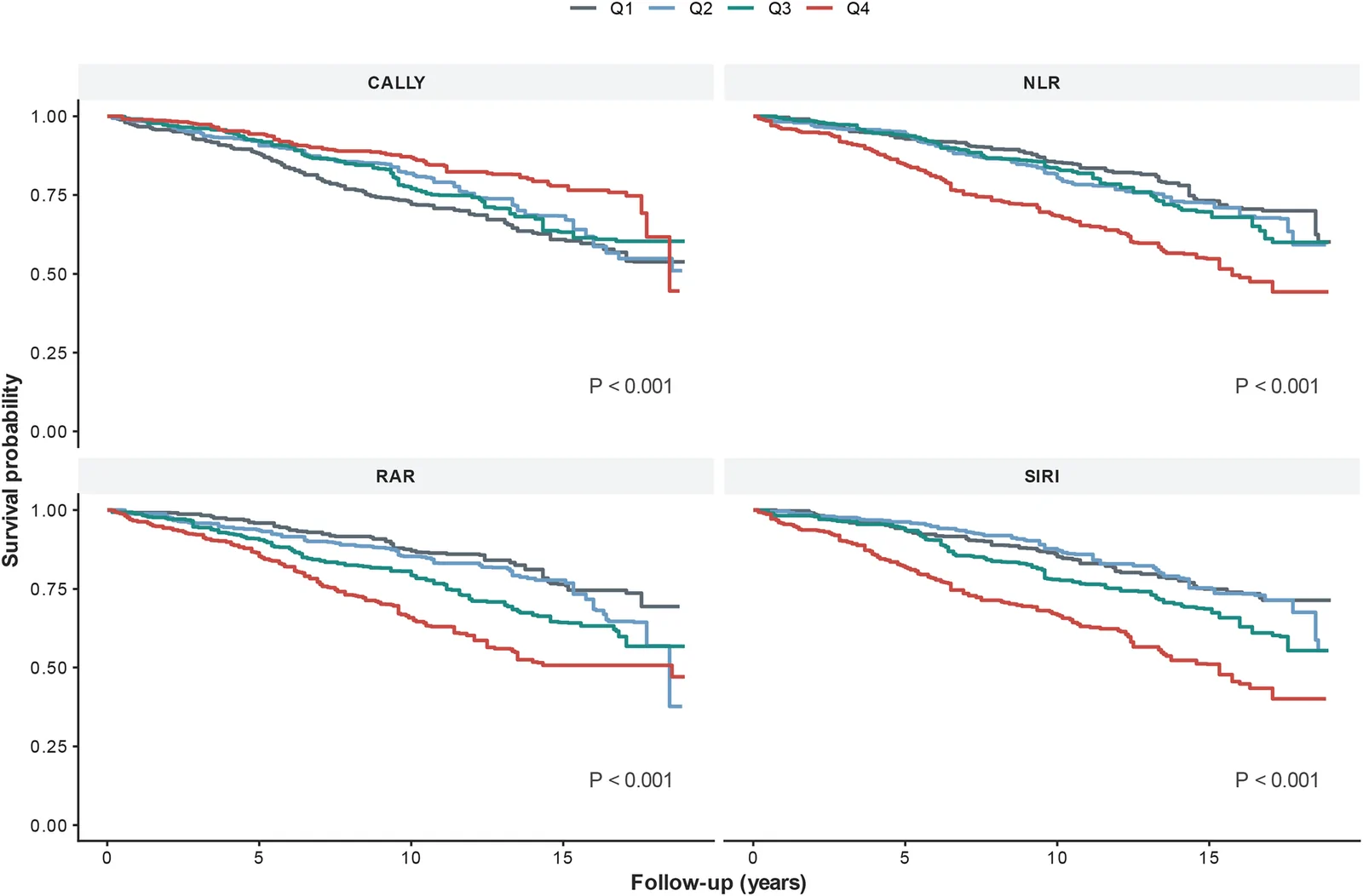
Kaplan–Meier curves for all-cause survival according to quartiles of inflammatory indices in patients with rheumatoid arthritis. Survival curves stratified by quartiles of CALLY, NLR, RAR, SIRI.

### Fully adjusted survey-weighted nonlinear associations between inflammatory indices and all-cause mortality

Fully adjusted survey-weighted restricted cubic spline analyses showed significant overall associations of CALLY, RAR, LMR, and NLR with all-cause mortality among participants with RA (Figure 5; Supplementary Table S9; all *P* for overall association, FDR < 0.001). Significant nonlinear associations were observed for CALLY (*P* for nonlinearity, FDR < 0.001), RAR (*P* for nonlinearity, FDR = 0.019), and LMR (*P* for nonlinearity, FDR < 0.001), whereas the association between NLR and mortality was approximately linear (*P* for nonlinearity, FDR = 0.437). Relative to the survey-weighted median, mortality risk decreased sharply with increasing CALLY at lower values and then remained comparatively stable. Higher RAR values were associated with an increasing mortality risk, particularly above the reference value. LMR showed a U-shaped association, with the lowest estimated risk at intermediate values. In contrast, NLR showed a positive, approximately linear association with all-cause mortality.

**Figure 5:**
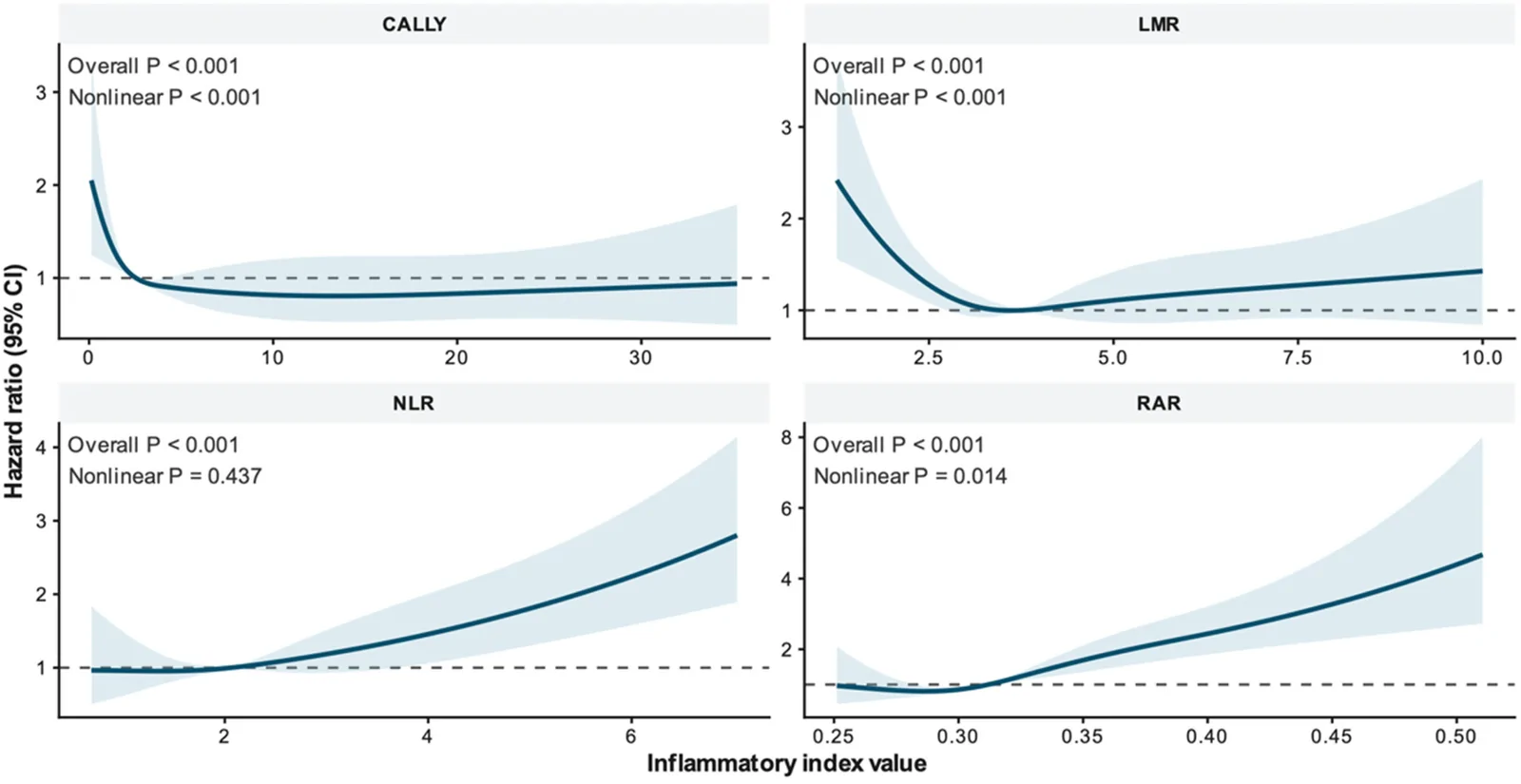
Restricted cubic spline analysis of the associations between inflammatory indices and all-cause mortality. Restricted cubic spline curves illustrating the associations of CALLY, RAR, LMR, and NLR with all-cause mortality.

### Baseline characteristics of the local hospital cohort

A total of 1,718 non-RA health-examination participants and 1,754 patients with RA were included in the local hospital cohort (Table 3). Compared with non-RA health-examination participants, patients with RA were significantly older and exhibited higher HbA1c levels but lower total cholesterol levels (*P* < 0.001). The proportion of females was significantly higher among patients with RA than among non-RA health-examination participants (60.1% vs. 46.4%, *P* < 0.001). Regarding inflammatory indices, RA patients demonstrated significantly higher levels of NLR, PLR, SII, SIRI, PIV, RAR and NAR, whereas LMR and CALLY were significantly lower than those in non-RA health-examination participants (*P* < 0.001). The independent hospital-based cohort showed directionally consistent patterns for several RA-related inflammatory indices compared with those observed in NHANES, although the magnitude of the between-group differences varied between the two cohorts.

**Table 3:** Baseline characteristics and inflammatory indices of the local hospital cohort.

Variable	level	Non-RA	RA	*p*
n		1,718	1,754	
Sex	Female	798 (46.4)	1,054 (60.1)	<0.001
	Male	920 (53.6)	700 (39.9)	
Age		47.00 (38.00, 56.00)	62.00 (54.00, 71.00)	<0.001
HbA1c		5.57 (5.30, 5.90)	6.30 (5.70, 7.30)	<0.001
TC		5.08 (4.49, 5.73)	4.82 (4.06, 5.53)	<0.001
NLR		1.68 (1.33, 2.13)	2.53 (1.78, 3.88)	<0.001
PLR		113.46 (91.57, 139.84)	130.41 (98.94, 174.05)	<0.001
LMR		5.87 (4.62, 7.24)	3.68 (2.64, 4.88)	<0.001
SII		367.84 (270.88, 492.57)	583.98 (354.22, 956.26)	<0.001
SIRI		0.55 (0.39, 0.79)	1.22 (0.74, 2.03)	<0.001
PIV		119.94 (80.63, 180.32)	277.65 (149.14, 509.52)	<0.001
RAR		0.28 (0.27, 0.30)	0.35 (0.31, 0.40)	<0.001
NAR		0.07 (0.06, 0.09)	0.12 (0.08, 0.16)	<0.001
CALLY		10.70 (5.10, 20.91)	2.82 (0.69, 7.74)	<0.001

Continuous variables are presented as median [interquartile range (IQR)], and categorical variables are presented as *n* (%).

In multivariable logistic regression analyses adjusted for age, sex, HbA1c, and total cholesterol, higher quartiles of NLR, PLR, SII, SIRI, PIV, RAR and NAR were generally associated with progressively increased odds of RA, with particularly pronounced associations observed for RAR, SIRI, and PIV. In contrast, higher quartiles of LMR and CALLY were consistently associated with lower odds of RA, showing clear inverse dose–response patterns (Figure 6). These adjusted findings provided additional evidence of associations between systemic inflammatory indices and RA after multivariable adjustment in the local hospital cohort.

**Figure 6:**
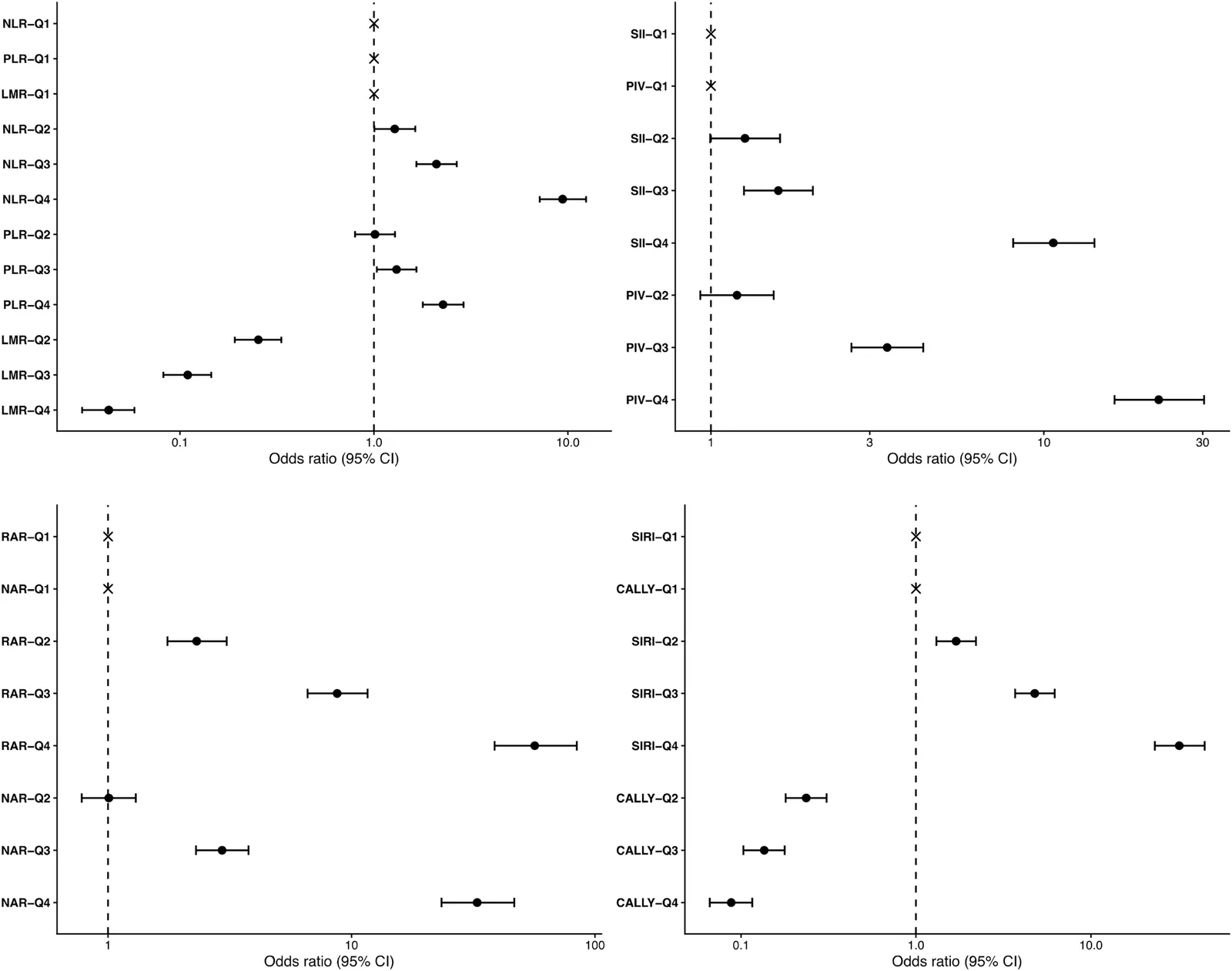
Multivariable associations between inflammatory indices and rheumatoid arthritis in the local hospital cohort.

## Discussion

In this study, we evaluated the associations of multiple systemic inflammatory indices with prevalent RA and all-cause mortality using nationally representative NHANES data and an independent hospital-based cohort. In the primary fully adjusted quartile analyses, higher RAR and lower CALLY were associated with prevalent RA after multivariable adjustment, and both indices remained associated with all-cause mortality among participants with RA after FDR correction. Complementary continuous and restricted cubic spline analyses also supported an inverse association between total bilirubin and prevalent RA and indicated associations of NLR and LMR with mortality; however, these latter findings were not uniformly retained in the primary quartile analyses and should therefore be interpreted as exploratory. Overall, RAR and CALLY showed the most consistent associations across the cross-sectional and prognostic analyses, whereas the other indices may reflect additional inflammatory, immune, and nutritional pathways that warrant prospective validation.

Among the evaluated indices, RAR and CALLY showed consistent associations with both RA and all-cause mortality, indicating that they may capture clinically relevant inflammatory and nutritional disturbances across different disease outcomes. RAR, calculated as the ratio of RDW to albumin, integrates information related to systemic inflammation, oxidative stress, erythrocyte heterogeneity, and nutritional status (16). Elevated RDW has been linked to inflammatory activation and impaired erythrocyte homeostasis, whereas reduced albumin commonly reflects chronic inflammation and diminished nutritional reserve (17, 18). Accordingly, a higher RAR may represent a more adverse systemic inflammatory–nutritional profile associated with both the presence of RA and poorer prognosis. By contrast, CALLY combines albumin, lymphocyte count, and CRP and therefore reflects nutritional status, immune competence, and inflammatory burden (19). Its inverse associations with RA and mortality suggest that higher CALLY values may indicate a more favorable systemic inflammatory and nutritional condition rather than a direct protective effect. Notably, albumin is incorporated into both RAR and CALLY, underscoring its potential importance as a marker linking inflammation, nutritional status, and clinical outcomes in RA (20).

In analyses of prevalent RA, total bilirubin showed an inverse association in the fully adjusted continuous and restricted cubic spline models, although the Q4-versus-Q1 association did not remain statistically significant after FDR correction. Bilirubin is increasingly recognized as an endogenous antioxidant with anti-inflammatory properties. Previous studies have suggested that bilirubin may reduce oxidative stress and modulate immune responses, thereby contributing to a more favorable inflammatory profile (21, 22). The observed inverse association is therefore biologically plausible; however, the cross-sectional design precludes causal inference or conclusions regarding whether bilirubin directly influences RA development. Bilirubin was not consistently associated with all-cause mortality among participants with RA, suggesting that its relevance may differ between RA prevalence and long-term prognosis. Further longitudinal studies are needed to determine whether bilirubin has a biological role in RA or primarily reflects systemic oxidative and inflammatory status.

Regarding prognosis, associations of NLR and LMR with all-cause mortality were observed primarily in the continuous and restricted cubic spline analyses, rather than uniformly across the primary FDR-adjusted quartile contrasts. NLR reflects the balance between neutrophil-mediated inflammatory activity and lymphocyte-mediated immune regulation, and elevated NLR has been widely associated with poorer outcomes in chronic inflammatory and malignant diseases (23, 24). In the present study, higher NLR was associated with greater all-cause mortality, suggesting that a shift toward neutrophil-predominant inflammation may reflect a more adverse systemic immune state. Conversely, higher LMR was associated with better survival. As LMR reflects the relative balance between lymphocytes and monocytes, lower values may indicate impaired adaptive immunity together with enhanced monocyte-related inflammatory activity. Collectively, these findings suggest that disturbances in innate and adaptive immune balance may be relevant to long-term outcomes in RA, although the observational design does not establish a causal relationship.

Restricted cubic spline analyses indicated that several associations varied across the observed ranges of inflammatory indices. For prevalent RA, significant nonlinear associations were observed for RAR and CALLY, whereas the inverse association with total bilirubin was approximately linear. In the survival analyses, CALLY, RAR, and LMR showed nonlinear associations with all-cause mortality, whereas the association for NLR was approximately linear. These exploratory findings suggest that assuming strictly linear associations may overlook potentially relevant exposure-outcome patterns. However, because specific inflection points were not formally identified or validated, these results should be interpreted as evidence of nonlinearity rather than definitive threshold effects.

The independent hospital-based cohort provided additional evidence for the reproducibility of the RA-related findings. Several inflammatory indices, particularly RAR and CALLY, showed directionally consistent patterns with those observed in NHANES, although the magnitude of the between-group differences was generally greater in the hospital cohort. This variation may partly reflect differences in population selection, case ascertainment, and laboratory settings between the two cohorts. In addition, the hospital-based RA patients and non-RA health-examination participants differed substantially in age, sex distribution, and metabolic characteristics. Such differences may have increased phenotypic separation between groups and introduced selection or spectrum bias. Accordingly, the hospital-based findings should be interpreted as independent, directionally consistent replication of several RA-related associations rather than as evidence of identical effect sizes across populations. Although the hospital-based analyses were adjusted for available demographic and metabolic characteristics, residual confounding cannot be excluded, particularly given the substantial baseline differences between the two groups. Therefore, the relatively strong associations observed in the hospital cohort should be interpreted with caution.

From a clinical perspective, the inflammatory indices evaluated in this study can be readily calculated from routine hematological and biochemical measurements, making them inexpensive and broadly accessible. Their use may provide additional information beyond individual laboratory parameters when assessing systemic inflammatory and nutritional status in patients with RA. In particular, RAR and CALLY may be useful candidates for further evaluation because they were consistently associated with both RA and all-cause mortality. However, these indices should not currently be used as stand-alone screening or prognostic tools. Their incremental value over established clinical factors, disease activity measures, and conventional biomarkers requires confirmation in prospective studies before routine clinical implementation.

Several limitations should be acknowledged. First, the cross-sectional nature of the NHANES analysis precludes causal inference, and reverse causation cannot be excluded because established RA and its treatment may influence inflammatory indices. RA status was based on self-reported physician diagnosis and reported arthritis type rather than medical-record verification, which may have introduced misclassification. Second, inflammatory indices were derived from single measurements and may not reflect long-term inflammatory status. Detailed RA-specific information, including disease duration, disease activity, autoantibody status, treatment, and glucocorticoid exposure, was not consistently available, while acute infection and nutritional status may also influence these markers. Third, despite multivariable adjustment, residual confounding remains possible, and the complete-case approach may have introduced selection bias. Several inflammatory indices also share common laboratory components, and subgroup and nonlinear analyses involving multiple comparisons should be considered exploratory. Finally, the retrospective hospital-based replication cohort differed from NHANES in population characteristics and laboratory settings, and residual confounding and selection bias cannot be excluded. Therefore, these findings should be interpreted cautiously and require confirmation in prospective multicenter studies with more detailed clinical information and longitudinal follow-up.

## Conclusion

In conclusion, systemic inflammatory indices showed distinct associations with prevalent self-reported rheumatoid arthritis and all-cause mortality among participants with established RA. RAR and CALLY demonstrated the most consistent findings across the primary fully adjusted quartile models and complementary continuous and spline analyses: higher RAR and lower CALLY were associated with both prevalent RA and mortality. Total bilirubin showed an inverse continuous association with prevalent RA, whereas NLR and LMR showed mortality-related patterns primarily in the continuous and spline analyses; these secondary findings should be interpreted as exploratory. Routinely available inflammatory and immune-nutritional indices may complement the characterization of systemic inflammatory burden in RA. However, the observational design precludes causal inference, and prospective studies with external validation are needed before these indices can be considered for clinical risk assessment or prognostic use.

## Data Availability

The raw data supporting the conclusions of this article will be made available by the authors, without undue reservation.
