# Supplementary material for: Associations of systemic inflammatory indices with rheumatoid arthritis and all-cause mortality: a NHANES study with independent hospital-based replication
Source: Front Med (Lausanne). 2026 Oct 2;13:1968785. doi: 10.3389/fmed.2026.1968785 (PMC13672207; doi:10.3389/fmed.2026.1968785)
Supplement: Supplementary file 1 [file Image1.pdf]

NHANES 2001-2002 to 2009-2010

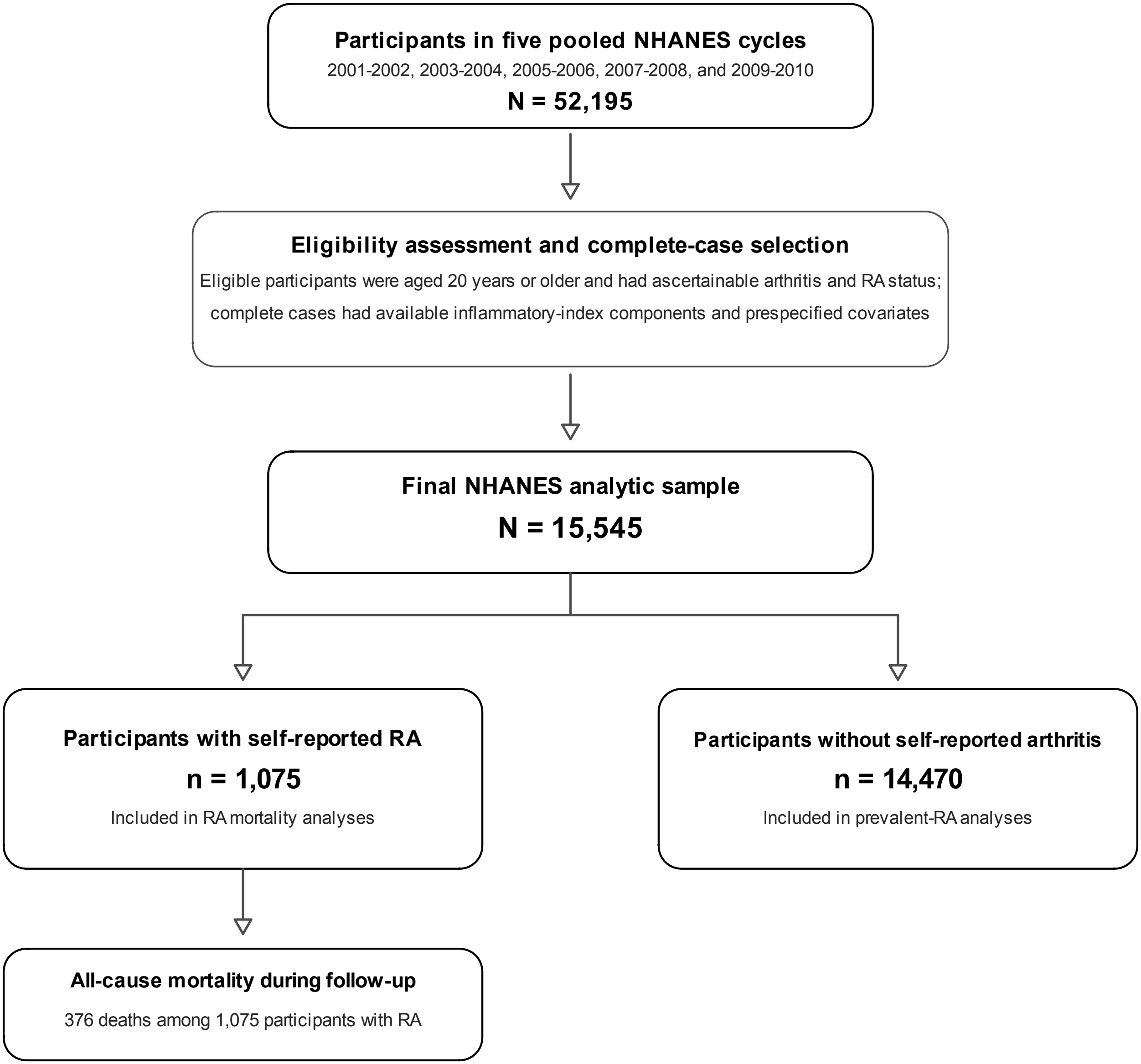

RA, rheumatoid arthritis. Counts are unweighted participant counts.
