# Supplementary material for: Associations of systemic inflammatory indices with rheumatoid arthritis and all-cause mortality: a NHANES study with independent hospital-based replication
Source: Front Med (Lausanne). 2026 Oct 2;13:1968785. doi: 10.3389/fmed.2026.1968785 (PMC13672207; doi:10.3389/fmed.2026.1968785)
Supplement: Supplementary file 2 [file Image2.pdf]

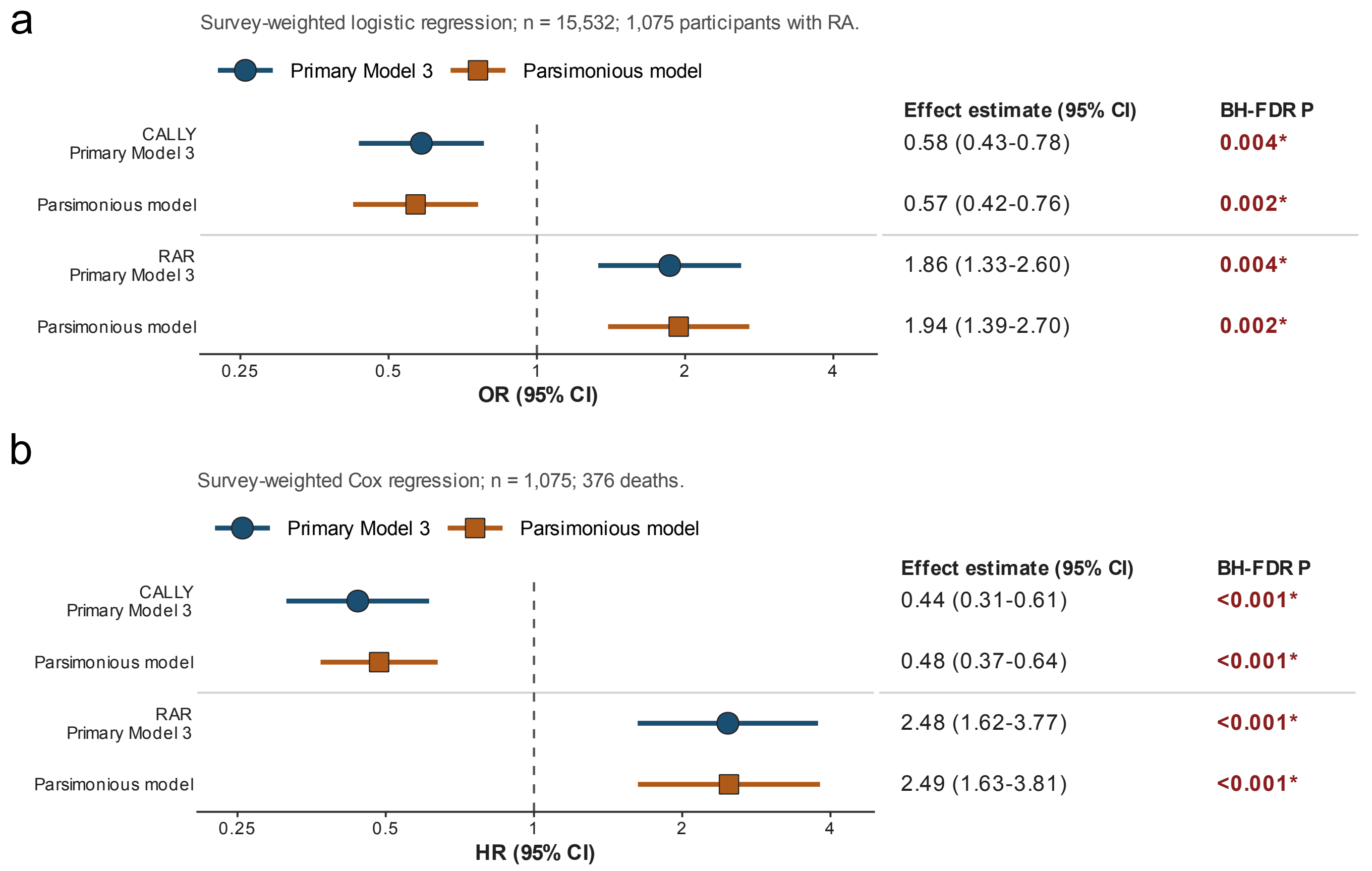
a
Survey-weighted logistic regression; n = 15,532; 1,075 participants with RA.
Primary Model 3
Parsimonious model
Effect estimate (95% CI)
BH-FDR P
CALLY
Primary Model 3
0.58 (0.43-0.78)
0.004*
Parsimonious model
0.57 (0.42-0.76)
0.002*
RAR
Primary Model 3
1.86 (1.33-2.60)
0.004*
Parsimonious model
1.94 (1.39-2.70)
0.002*
0.25
0.5
1
2
4
OR (95% CI)
b
Survey-weighted Cox regression; n = 1,075; 376 deaths.
Primary Model 3
Parsimonious model
Effect estimate (95% CI)
BH-FDR P
CALLY
Primary Model 3
0.44 (0.31-0.61)
<0.001*
Parsimonious model
0.48 (0.37-0.64)
<0.001*
RAR
Primary Model 3
2.48 (1.62-3.77)
<0.001*
Parsimonious model
2.49 (1.63-3.81)
<0.001*
0.25
0.5
1
2
4
HR (95% CI)
