# Supplementary material for: Associations of systemic inflammatory indices with rheumatoid arthritis and all-cause mortality: a NHANES study with independent hospital-based replication
Source: Front Med (Lausanne). 2026 Oct 2;13:1968785. doi: 10.3389/fmed.2026.1968785 (PMC13672207; doi:10.3389/fmed.2026.1968785)
Supplement: Supplementary file 3 [file Image3.pdf]

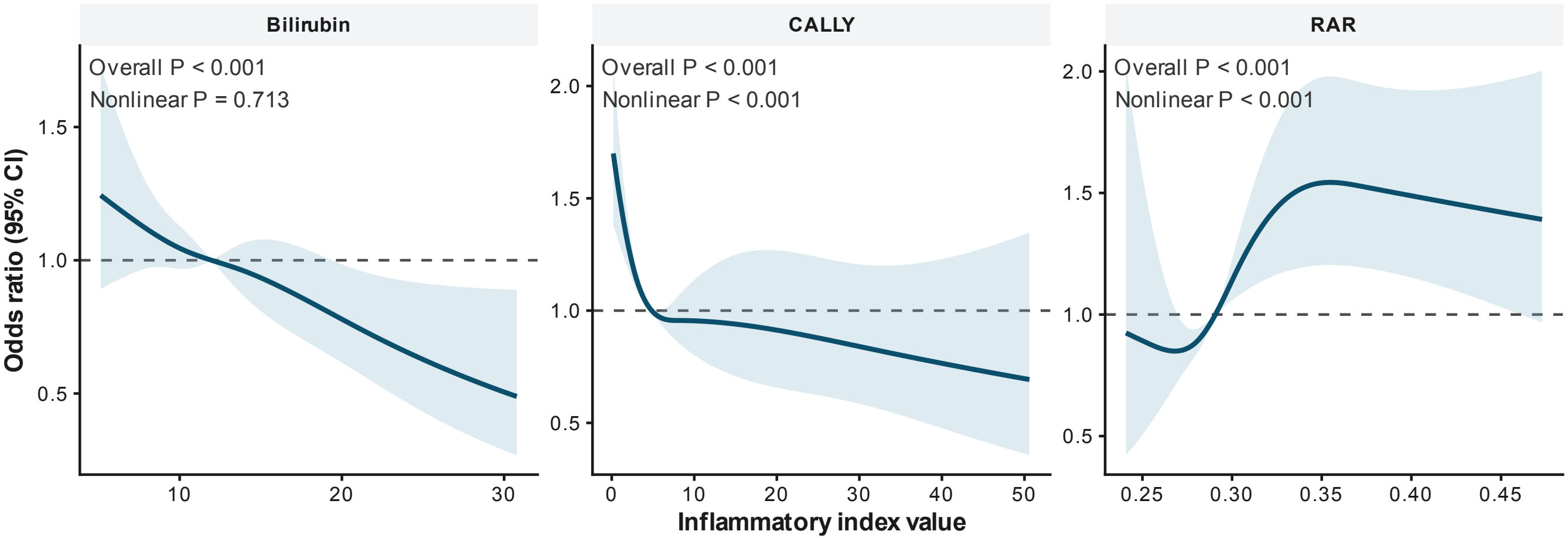
Bilirubin
CALLY
RAR
Overall P < 0.001
Nonlinear P = 0.713
Overall P < 0.001
Nonlinear P < 0.001
Overall P < 0.001
Nonlinear P < 0.001
Odds ratio (95% CI)
Inflammatory index value
0.5
1.0
1.5
2.0
10
20
30
0
40
50
0.25
0.30
0.35
0.40
0.45
