# Supplementary material for: Associations of systemic inflammatory indices with rheumatoid arthritis and all-cause mortality: a NHANES study with independent hospital-based replication
Source: Front Med (Lausanne). 2026 Oct 2;13:1968785. doi: 10.3389/fmed.2026.1968785 (PMC13672207; doi:10.3389/fmed.2026.1968785)
Supplement: Supplementary file 4 [file Image4.pdf]

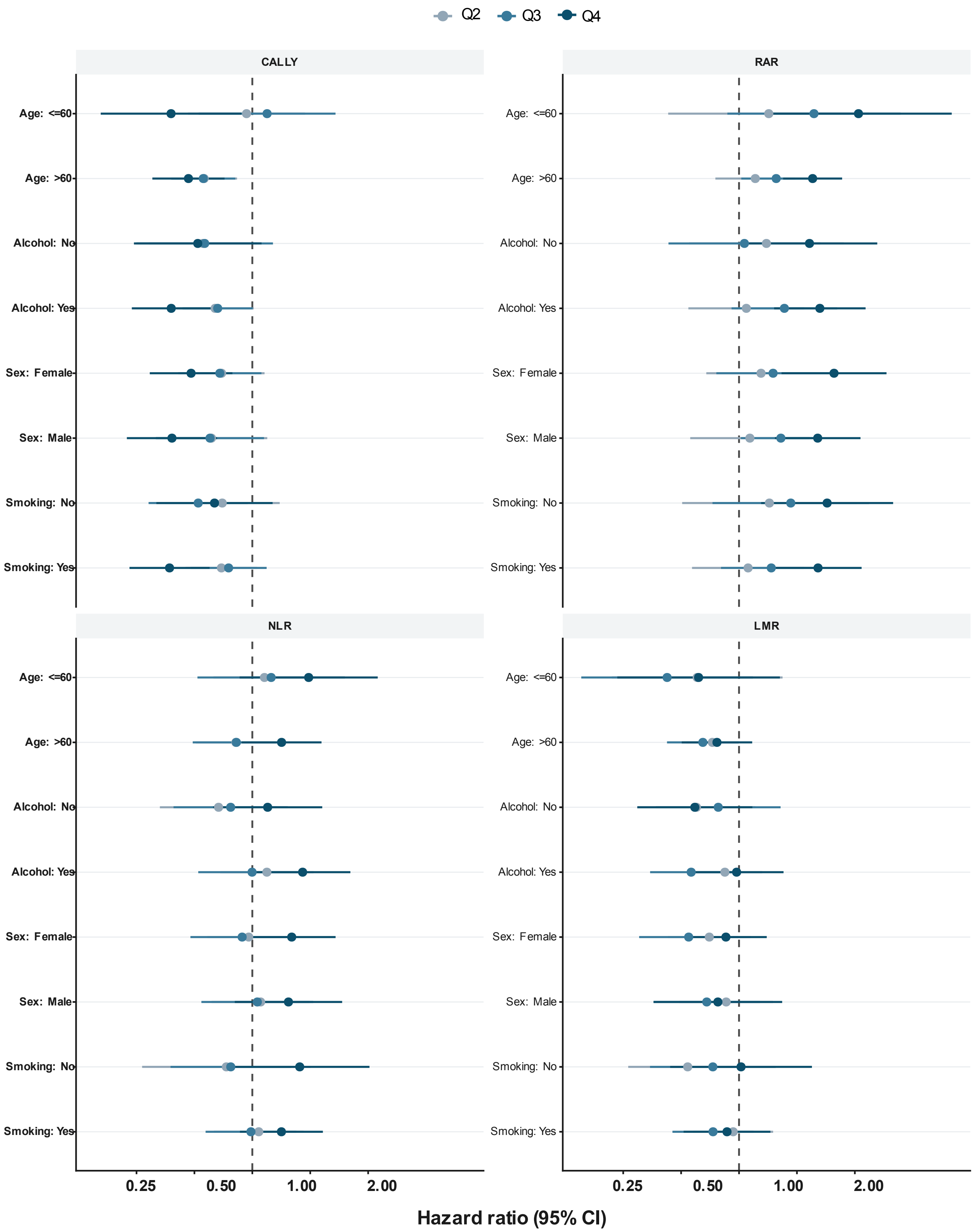

Q2
Q3
Q4
CALLY
RAR
NLR
LMR
Age: <=60
Age: >60
Alcohol: No
Alcohol: Yes
Sex: Female
Sex: Male
Smoking: No
Smoking: Yes
0.25
0.50
1.00
2.00
Hazard ratio (95% CI)
